# Advance directives in France: do junior general practitioners want to improve their implementation and usage? A nationwide survey

**DOI:** 10.1186/s12910-019-0358-x

**Published:** 2019-03-18

**Authors:** Sidonie Hubert, Sarah Wainschtein, Albane Hugues, Caroline Schimpf, Thècle Degroote, Kelly Tiercelet, Marc Tran, Cédric Bruel, Francois Philippart, for the REQUIEM group (REQUIEM: Research/Reflexion on End of life support QUality In Everyday Medical practice)

**Affiliations:** 1 Internal medicine Unit, Groupe hospitalier Paris Saint Joseph, Paris, France; 2 Medical and surgical intensive care unit, Groupe hospitalier Paris Saint Joseph, Paris, France

**Keywords:** Advance directives, Living wills, Patient autonomy, General practitioner, Medical education

## Abstract

**Background:**

The doctor-patient relationship has evolved to respect “the autonomy and patients’ rights”. One of the cornerstones in such autonomy is the opportunity for patients to draw living wills, also known as advance directives (AD). However, information about AD available to patients remains scarce largely due to the lack of involvement of General practitioners for several reasons. The aim of our study was to evaluate current general practitioner residents’ (GPR) behavior concerning their role in informing their patients about AD.

**Method:**

We built a French nationwide survey from GPR class of 2012 to 2014.

**Results:**

Two thousand three hundred ten residents completed our survey (21.1% of the total population of GPR during the period). 89.8% declared their willingness to offer patients the opportunity of writing AD. When asked about the usefulness of AD, 73.6% of residents responded that these are a suitable help for patients, but 19.7% considered that AD are essentially geared towards frail patients. Among residents who want to inform patients about AD (*n* = 2075), 14.7% wanted to involve all patients. Only 20.5% thought that elderly people should be systematically informed about AD. When the question involves other frail people in various disease areas, information seems relevant for 60.1% of GPR considering patient with cancer or malignant hematologic disease and for 56.2% about patients affected by neurodegenerative disease. When considering the routine use of AD, 20.5% of GPR would take them into account only if they are in agreement with the patient’s decision.

**Conclusions:**

The results of the survey indicate that GPR would rather choose to decide who should be informed about AD, and when to take AD into account for ethical concerns.

**Electronic supplementary material:**

The online version of this article (10.1186/s12910-019-0358-x) contains supplementary material, which is available to authorized users.

## Background

Scientific advances in recent decades have led to an improvement in the prognosis of chronic organ failure, an increase in survival in most severe diseases, and an increase in the life expectancy of the general population. At the same time, the proportion of the frail population [1, 2] has also increased, with potentially resulting severe disabilities leading to a questionable benefit of therapeutic intensification, and the consideration that quality rather than length of life should in some cases prevail. Favoring welfare to therapeutic efficacy is a difficult decision, particularly when the patient view is not available.

Aiming to avoid obstinacy, and to emphasize the importance of patients wishes, scientific and clinical developments have led to progressive changes in laws in many countries around the world, centered on the possibility for everyone to express their preferences regarding the intensity of care they wish to benefit [3]. The notion of advance directives (AD) was created in the United States in the late 1960s, in accordance with the principle of self-determination (Patient Self Determination Act) and was progressively adopted throughout the world [3, 4]. In France, AD were incorporated into the law in 2005 (Leonetti’s law relating to Patients’ rights and to the End of life) [5]. This first version of the law was not legally binding for physicians, but should “*be taken into account*” for ethical reflections when the patient is unable to express his opinion. There was, however, a priority of medical practice over patients’ preferences, and physicians were allowed not to take AD into account because of uncertainty in sustainability of patients’ choices or because of apparent discrepancy between patients’ status and AD, suggesting that the wishes expressed by the patient were not taken with sufficient knowledge, and AD must be less than three years-old to be valid.

The objective of AD is to allow patients to become an actor for their end-of-life medical health and interventions, by expressing in advance their wishes regarding intensification therapy, but also to inform physicians about their personal beliefs, values, and preferences [6]. In order to allow the drafting of such a document, patients should be informed of their existence, and assisted with the drafting of AD (understanding the pathologies, their evolution and their prognosis and the possible consequences of expressed choices).

Despite all these official measures, the proportion of patients processing advance directives remains low [3, 6–8]. The absence of practical application of the law would be linked to the lack of information of the patients concerning the existence and use of these guidelines, secondary to a lack of knowledge of the law by the clinicians [9, 10], but also to a willingness not to address the end-of-life problem with their patients [9, 11]. Despite recent educational changes in many countries [12], no significant modification of proportion of patients with AD was observed.

The aim of our study is to evaluate how the new generation of clinicians are dealing with AD, and their involvement in implementing them with their patients.

## Methods

### Questionnaire

The questionnaire consisted of 37 questions divided in four parts (Additional file 1: Survey): information about participants; knowledge of the law about advance directives; willingness to take part in advance directives implementation; use of AD in potential critical situations. The survey was anonymous and was filled out on a voluntary basis.

The questionnaire was hosted by a specific site: Survey Monkey® and was available online from September 2014 to November 2015.

### Population

In France medical studies are constituted of two different periods: a first one (six years) of initial and common formation and a second one (“*internat*”) during which they choose a medical or surgical specialty, and work in different wards to lean specificity of their future job. This is probably close to “fellowship” in the US. This period lasts three years for general practitioners. This was our target population. The General practitioner resident (GPR) internship consists of six, 6-months internships either in hospital departments or in GP’s office. Our population of interest was constituted of the whole GPR residents. The population was contacted using mailing lists obtained by GP departments of regional universities, mailing list of students’ groups and associations and mailing list of residents’ unions. No official promotion of the study was available but two authors (SW and AH) have sent mails to the head of GPR in each university. Three reminders mails were sent to each group of GPR (one group per City/University). Individual mails were also sent when they were available. We try to contact educational leaders without any answer.

### Ethics

The questionnaire was anonymous and was filled out on a voluntary basis. As this was a voluntary survey, we examined participation after the survey was introduced. Due to its broadcasting on the Internet, an authorization of the CNIL (National Commission of Informatics and Liberties) was solicited and obtained.

According to French law, this survey was a non-interventional study. Its non-interventional nature does not require submission to a mandatory ethics committee. As such we have no IRB number. However, the study was presented to an ethic commission in our hospital, which did not request any changes. It did not require consent but simply “non-opposition” from participants. Since the survey was done remotely, we add before the start of the survey a first paragraph indicating the aim of the survey and the possibility of the reader not to take part to the survey. We also indicated that data obtained in the survey will be analyzed and published in a scientific journal. Participants were considered not to oppose the study if they answered the questionnaire without necessity to collect a formal agreement (“non-opposition”).

### Statistical analysis

Data were expressed as mean and standard deviations or, if appropriate, median and confidence intervals as a function of response disparity.

## Results

### Population

**Fig. 1:**
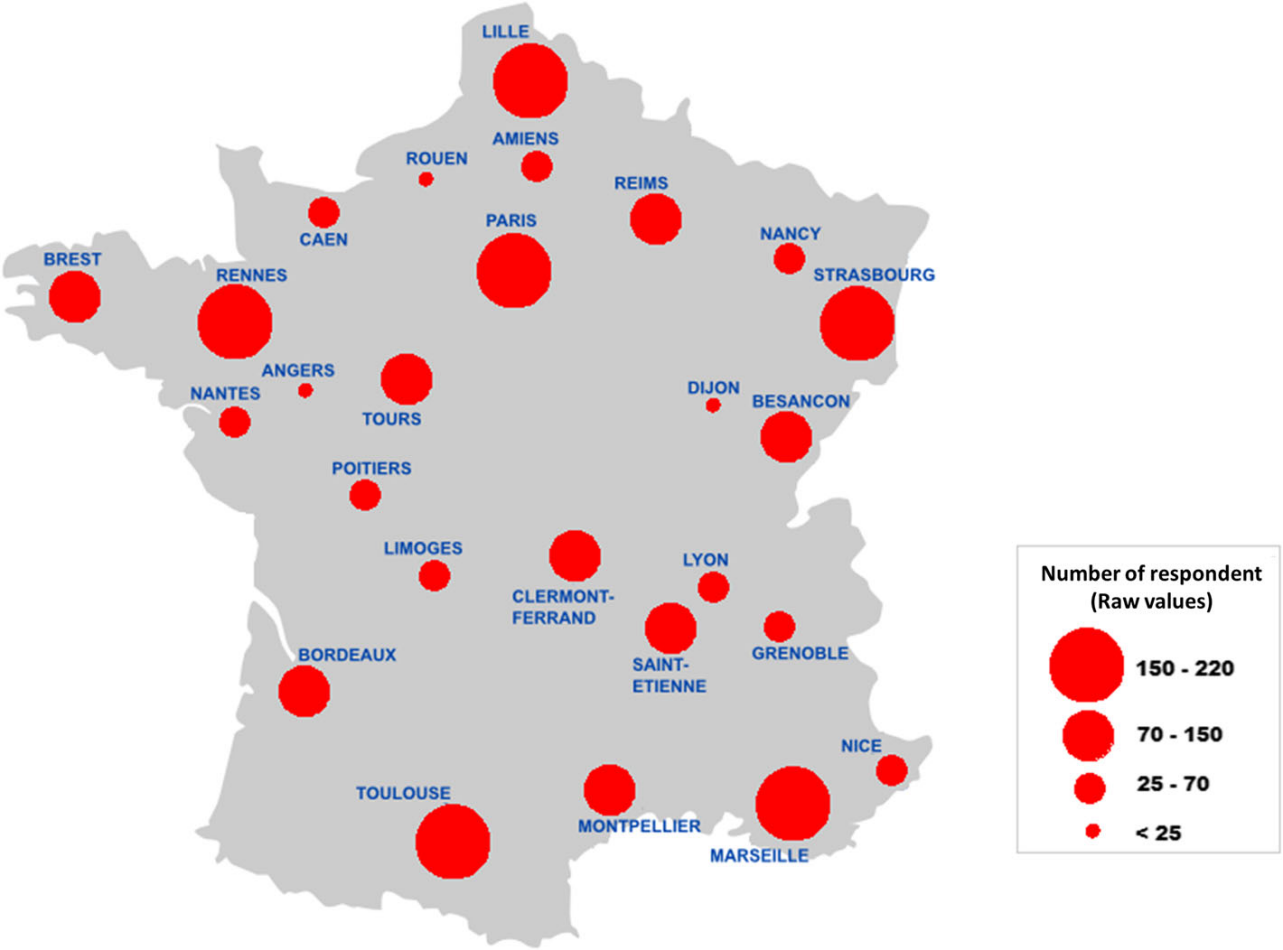
Raw repartition of respondent around France

2310 residents filled out our survey over a total of 10,942 GPR solicited during the study period (21.1%). As indicated in the emails, GPR who did not want to answer the questionnaire did not have to provide any reasons. Females represent 74.4% of participants and the mean age was 27.5 (+/− 5.5). Every French regions were represented (summarized in Table 1 and Fig. 1). The distribution of GPR related to their seniority was 26.9% in first year, 31.8% in second year and 30.2% in third year of residency. Of all the GPR, 69.4% already worked in a GP office.

**Table 1:** Best and worse responding cities

City	Number of responders	Percentage of total responders	Number of residents in the city	Percentage of potential responders in the city
a. Best responding cities				
Besançon	101	4·4%	197	51·3%
Rennes	160	6·9%	318	50·3%
Toulouse	207	9%	475	43·6%
Strasbourg	172	7·4%	410	42·0%
Tours	99	4·3%	278	35·6%
b. Worse responding cities				
Rouen	11	0·5%	312	3·5%
Dijon	15	0·6%	282	5·3%
Nancy	22	1%	419	5·3%
Angers	23	1%	340	6·8%
Lyon	50	2·2%	542	9·2%

### Knowledge of AD

**Fig. 2:**
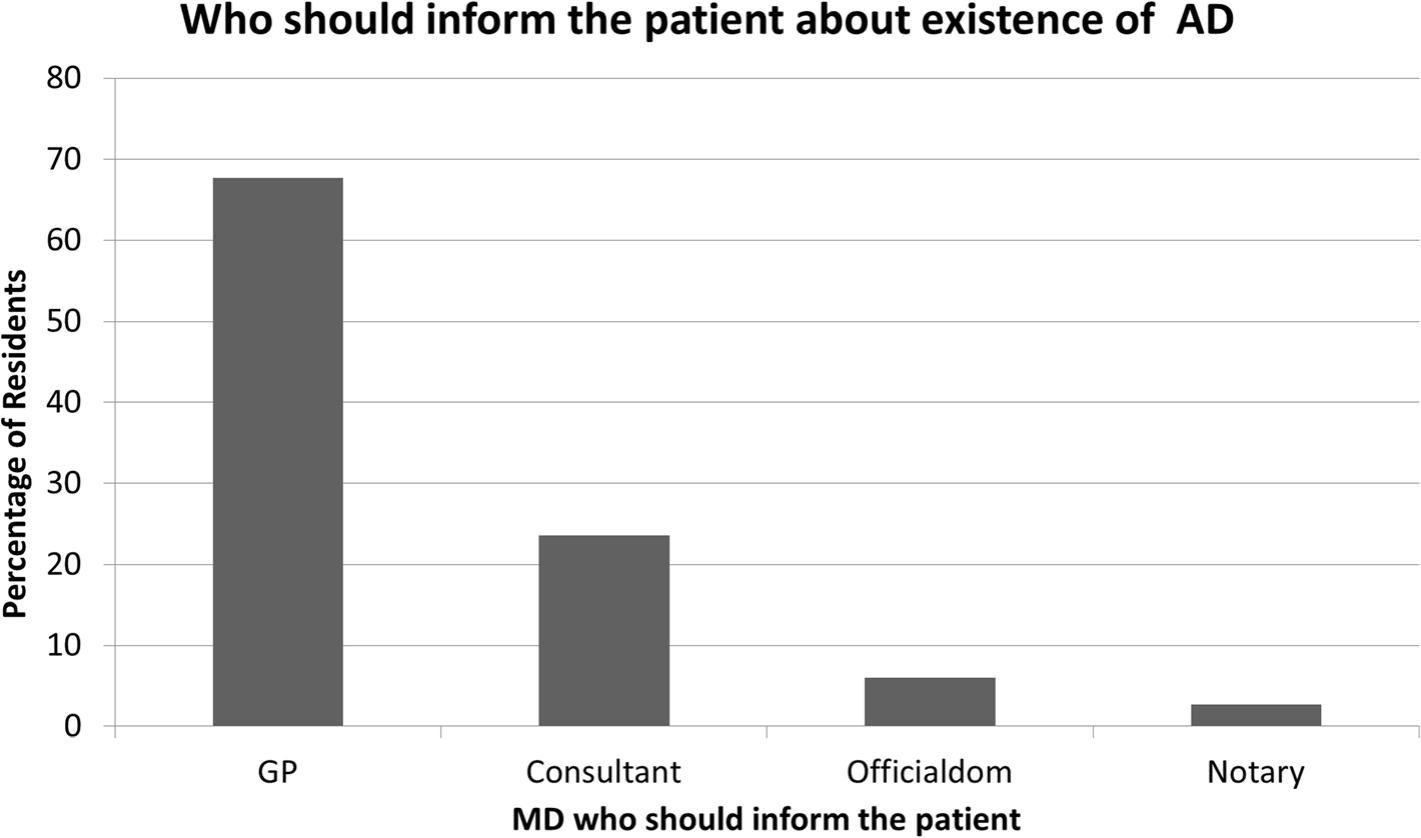
Who should inform patients about the existence of advance directives AD: Advance directives; GP: General practitioner

Among the responders, 94.7% declared they knew about advance directives. 91.6% of GPR considered that patients do not know anything about AD, but only 53.4% thought that patients need assistance in writing AD. For 67.7% General practitioner seemed to be the best MD to give patient information about AD (Fig. 2).

### Involvement in AD promotion

### 89.8% of participants declared they will offer their patients the opportunity to write AD

When considering the participants that did not want to give information about AD (10.2%, 235 participants), 72.3% declared that in their opinion, patients would not understand AD and the corresponding consequences.

### Patients that should benefit from information about AD

An overwhelming observation was that among GPR who wanted to suggest AD to their GP office patients (*n* = 2075), only 14.7% wanted to inform all their patients.

**Fig. 3:**
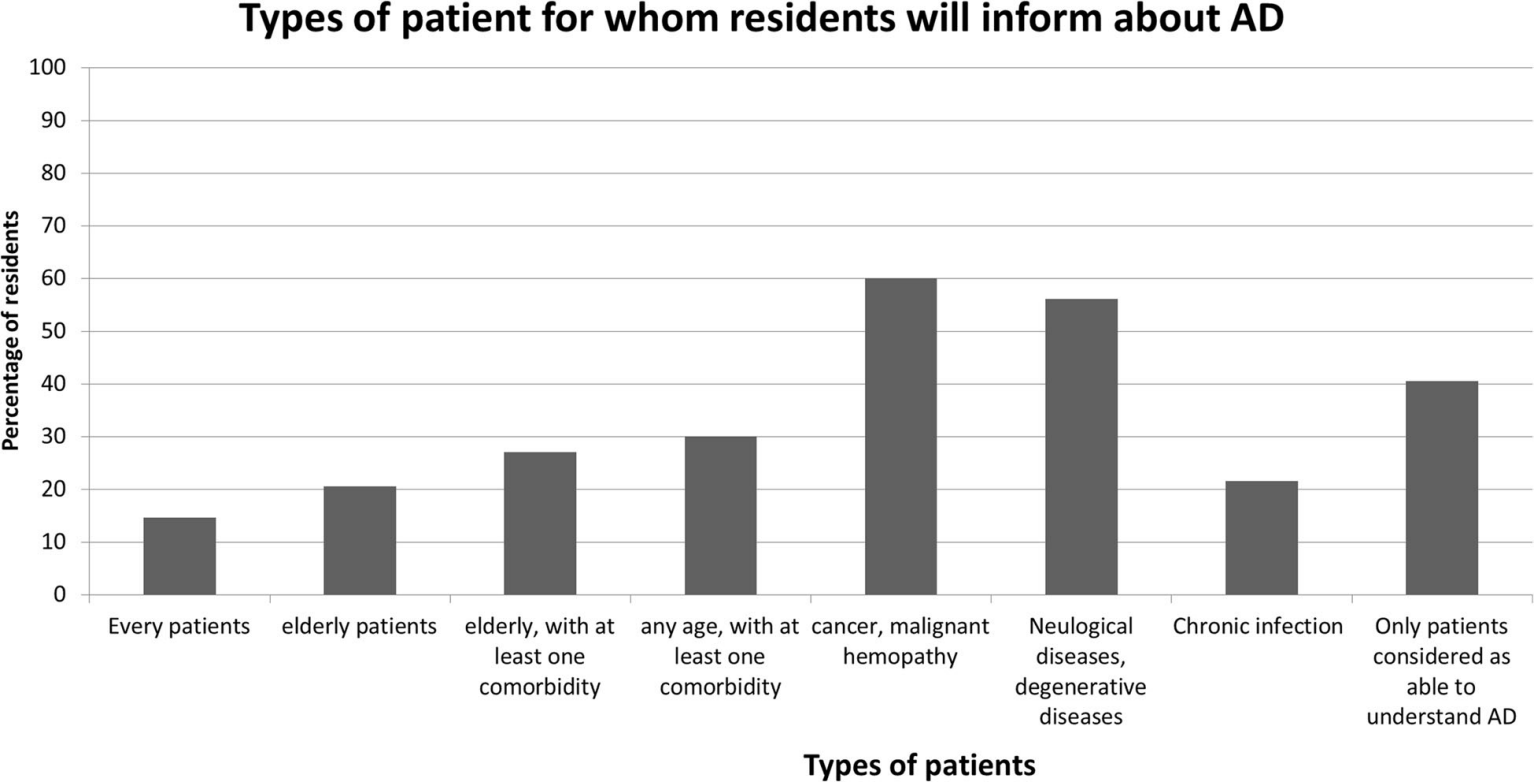
Which patients can benefit of information about advance directives AD: Advance directives

Specific and severe populations are not systematically considered as needing information about AD (Fig. 3). Such information should be given to patients with cancer or malignant hematologic disease for only 60.1% of GPR. A similar observation was made for patients affected by neurologic disorders, notably degenerative ones, 56.2% of GPR). The proportion of GPR that would offer patients information decreased when considering patients with numerous comorbidities independently of their age (30%) and elderly patients (20.5%).

### AD potential use

When asked about the usefulness of AD, 73.6% of residents responded that they are a suitable aid for patients and their autonomy in health. However, 19.7% considered AD are only adapted for “at risk” patients including frail patients, and 14.3% thought of AD as not adapted to clinical use.

Considering the use of AD in clinical practice, and having in mind the medical intensity consequences for their patients, only 17.9% would systematically take AD into account, 45.8% would accept “most often”, whereas AD should be interesting only if the patient is supposed to understand the consequences of his/her decision for 15.6% of participants. 20.5% acknowledged that they will use AD only if they agree with the decision of the patient and only 3 GPR declared that they would never take AD into account.

Likewise, in the case of a patient’s hospital admission, only 59.7% of GPR declared they would spontaneously transferred AD.

**Fig. 4:**
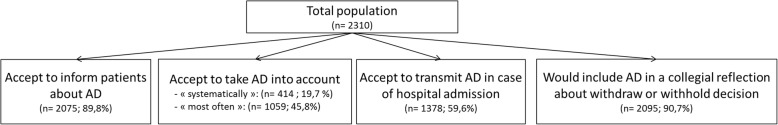
Repartition of the total population of the survey AD: Advance directive

To summarize (Fig. 4), 2075 of 2310 (89.8%) participants declared that they want to inform their patients about AD and eventually provide them help with drafting relevant AD. 1473 (63.7%) would take the AD into account (17.9% systematically and 45.8% “most often”). Considering the transfer of AD in case of patient hospitalization, 59.7% (*n* = 1378) would agree to indicate the existence of AD before admission. At last, 2095 (90.7% of total population) would include such AD in a collegial discussion about withdraw or withhold decision, the remaining part of the participants considering AD not to have a place in these ethical discussions.

## Discussion

Our study finds an important willingness of junior clinicians in taking part with the implementation of ADs, both by informing about their existence and by their wish to help patients in the drafting of these ADs. Despite this enthusiasm, the medical paternalism, illustrated by personal definition of population that should be informed, and the selection of AD that should be taken into account, remains important and maybe worrisome. Whereas the autonomy of the person is at the heart of the physician’s function [13] and the possibility for everyone to write AD as recently underlined by law [5], the residents who willingly took part in our survey only want to inform part of their patients, that vary according to the participant, and more than one quarter reported that they would only consider AD if they felt the patient understood what he had written or agreed with the patient’s wishes.

The design of our survey does not allow for the understanding of the potential factors underlying choices of GPR, nonetheless, difficulties of approaching the subject of the severe pathology and end of life with patients probably count for a significant part in their decision to select patients requiring to be informed about AD [14]. Concerns about patients’ understanding of the prognosis of severe disease emphasized in our survey is another usual factor declared by clinicians, limiting the application of AD, and is frequently associated with the desire to favor therapeutic intensity [12, 15].

Beside information and assistance with AD, more than 40% of participants affirmed they would not immediately transfer AD to hospital physician in case of patient admission. The lack of potential transfer of AD from patients to hospital clinicians has been known for a long time [14], especially among young general practitioners [7]. Several justifications can be put forward to explain this relative reluctance: first concern about patients’ understanding of his own decisions mentioned above; second, consideration about the possibility for patient to change their opinion at the time of hospital admission [14, 16, 17], and third, there is in the medical corpus a part of uncertainty about the relevance of the AD [13, 15, 18].

In our study, a very small percentage of responders would “systematically take AD into account”. Such a decision could be questionable considering that according to the 2005 French law, physician should have taken AD into account. The survey was not designed to explain such observations, but we already know that until the modification of the ethical law in France (2016) many doctors considered that patients where not competent to decide what level of therapeutic intensity they should receive and affirm they would not apply these AD arguing of the impossibility to be sure the patient did not have change his/her mind since the AD were wrote, or considering the patient unable to take an “informed decision” due to the lack of information concerning ICU or surgery, etc. … On the other hand, many clinicians were much more afraid of legal consequences of a therapeutic withdrawal or withholding than of an excess in intensity of care. Moreover, despite large modification in the current law, weaknesses remain leading French physician to keep some distance from AD. In emergency situation, “stabilization of the patient condition” could (and probably should) be done before taking AD into account, at least in case of uncertainty about patient status. On the same way, many semantic points remain unanswered, for example: AD are often considered as wished for “end of life” situation, but except for chronic diseases, this notion is far unclear leading to an excess of aggressive treatment of severe conditions even in case of DNR wishes of the patient; another example could be the notion of “refusal of therapeutic relentlessness” which does not mean anything, leaving the clinician to choose when the treatment becomes futile. These points of weakness of the law, this supposed difference between the text and spirit of the law and the fear of not doing enough (in curative cares) are favoring intensity instead of comfort care in many situations.

These disturbing findings may be unraveled by the limitations of our study. First, this is a French study, not reflecting the modification of global medical population thinking, but the nationwide character of the survey gave us a broad range of opinions by physicians working in different conditions and social environment. Second, the response rate was only 21.1% of the total potential population. However, to our knowledge this is the largest survey involving GP resident, with a sex ratio close to that of the total population (mean percentage of women in the whole GPR population of interest during the study period: 62.9%) and a distribution according to the years of training which also corresponds to that of the overall population. Moreover, as participation was made on a voluntary basis the risk was to select the residents most interested in the subject. The stringent selection of patients and the frequent decision not to transfer AD in case of hospitalization would probably even be worse in the whole population, reinforcing our observation. Third, the lack of open responses does not allow us to know the justification for the answers given, notably concerning the selection of patients to be informed of the AD or the choice of the situations to be described in order to facilitate the drafting of AD by patients, but the observations obtained open up new perspectives for future surveys.

These observations lead us to believe that much remains to be done, notably in the education and training of young clinicians in order to improve the autonomy of patients, whose advance directives are only one manifestation. Nevertheless, the desire of residents to get involved in patient information about AD is an important factor in that these directives are less an official form than a reflection process involving the physician-patient relationship [19], at least in countries were the law is least restrictive.

## Conclusions

In conclusion, our study emphasized the large AD knowledge by residents during the last few years and their willingness to get involve in patient information and writing of such living wills. On the other hand, patient autonomy illustrated by patient selection and lack of transfer of AD in case of hospitalization seems to still be a limiting factor in optimal AD dissemination in the whole population and need further investigation and probably medical education.

## Additional file

Additional file 1: Survey. (DOCX 20 kb)

## Abbreviations

AD: Advance directives
GPR: General practitioner residents
