# Supplementary material for: Advance directives in France: do junior general practitioners want to improve their implementation and usage? A nationwide survey
Source: BMC Med Ethics. 2019 Mar 18;20:19. doi: 10.1186/s12910-019-0358-x (PMC6423804; doi:10.1186/s12910-019-0358-x)
Supplement: Supplementary file 1 — Survey. (DOCX 20 kb) [file 12910_2019_358_MOESM1_ESM.docx]

Survey

The following data constitute the whole translation of the French survey as used for the study.

- Year of birth: _________
- Sex : M F
- Location of your home university
- Semester (number of intership already done. Each intership is six months in a ward/office)
- Did you already worked in :
  - Internal medicine
  - Geriatric
  - Neurology
  - Oncology
  - Hematology
  - Emergency room
  - Intensive care
  - GP office
- When you are graduate where do you want to work :
  - GP office
  - GP office and hospital
  - Hospital
  - Retirement home

**Informations about advance directives**

- Did you ever heard about « advance directives »
- If yes,
  - When :
    - During initial formation
    - During fellowship formation
    - At the hospital
    - At GP office
    - In your family circle
  - Do you know where you can find official document about advance directives?
     No Yes

Advance directives allows the patient to express in advance his wishes regarding the therapeutic intensity that he wishes to benefit from, in the event that he is unable to formulate them

- In your opinion, should the treating physician suggest to the patient write AD?

Yes No

- Will you be offer your patients the opportunity of drafting advance directives?

Yes No

- If Yes, to which pateints ?
  - Any patient
  - Elderly patients
    - More than 70
    - More than 80
    - More than 85
    - More than 90
  - Elderly patients with :
    - One comorbidity (renal failure, heart failure, chronic respiratory failure…)
    - More than one comorbidity
  - Patients with one chronic organ failure whatever their age
  - Patients with more than one chronic organ failure whatever their age
  - Cancer patients
    - Only elderly ones
    - whatever their age
  - Patients with chronic arterial disease
  - Patients with neurological disease
  - Patients with genetically disease
  - Patients with degenerative disease
  - undernourished patient
  - Patients with chronic infectious diseases (HIV, viral hepatatis…)
  - Only patients who seems to be able to understand the implations of writing advance directives
- Do you think advance directives should be offered
  - To the patient him/herself
  - To his/her relatives
  - To another membre of patient’s
- Concerning the information about existence and opportunity to write AD, who should give patient information :
  - His/her GP?
  - Hospital physicians?
  - Retirement home director
  - Civil services?
- Concerning the drafting of advance directives: in your opinion, who should offer the patient to help him/her wrinting the AD?
  - His/her GP?
  - A consultant in case of specific disease?
  - Civil service
  - Notary
  - Someone else , explicit :
- Concerning the drafting of advance directives: when to offer to help the patient writing them?
  - During the first appointment
  - During a usual appointment
  - During a specific and planned appointment
  - Depends, function of peculiar situations (diagnosis of severe disease, recent hospital admission…)
- Who should hold advance directives?
  - GP of the patient
  - Other physicians (ex: consultant for a specific and severe disease, intensivists…)
  - One or more relatives
  - Trustworthy person of the patient
  - Notary of the patient
  - A specific and official website
  - Anywhere/someone else, explicit:
- If your patient has written AD
  - You would take them into account :
    - Never
    - Systematically
    - Most often
    - Only if you consider the patient to have understood the consequences of his/her decisions
    - Only if you agree with patients decisions / AD seems relevant (in accordance with patient status, …)
  - If your patient should require hospital admission
    - You would systematically transmit AD to the Ward/ICU?
    - You would transmit AD only if the ward/ICU asks you to do so?
- If you decide never to take AD into account it is because:
  - You think your point of view about relevant healthcare for a patient is better than his/her
  - You think the patients are probably not able to understand the consequences of their decisions / AD
- In case the patient refuse specific therapeutic intensification
  - You can reject AD because you think the patient can benefit from these therapeutic measures?
  - You can reject AD because you think the opinion of the patient will change after this specific treatment?
  - You can reject AD because you think you could have trouble with the law is all means have not been implemented?
- Overall, you believe :
  - AD are easy to access and to use for patients
  - AD are an appropriate response to patients’s desire for health autonomy
  - Access to AD shoud be modified to be simplified
  - AD should be promoted to the population by civil service because they still not reach the target population
  - AD should be promoted to the GP by civil service because they still not reach the target population
  - AD should be available for GP on the internet
  - AD should only be available for patients at risk of acute and severe complications
  - AD should be removed from French law
  - AD should be mandatory according to the law
- About quality and relevance of advance directives
  - In your opinion, patients can make their health choices alone

Yes No

- - If you think that the patient should makes his/her decisions regarding AD with a MD, this MD should be :
    - GP
    - A consultant (in case the patient suffer from a specific diseases)
    - Another physician

Do you think withdrawal and withholding decisions must be made after take AD into account?

Yes No
